# Challenges of Identifying Clinically Actionable Genetic Variants for Precision Medicine

**DOI:** 10.1155/2016/3617572

**Published:** 2016-04-06

**Authors:** Tonia C. Carter, Max M. He

**Affiliations:** ^1^Center for Human Genetics, Marshfield Clinic Research Foundation, Marshfield, WI 54449, USA; ^2^Biomedical Informatics Research Center, Marshfield Clinic Research Foundation, Marshfield, WI 54449, USA; ^3^Computation and Informatics in Biology and Medicine, University of Wisconsin-Madison, Madison, WI 53706, USA

## Abstract

Advances in genomic medicine have the potential to change the way we treat human disease, but translating these advances into reality for improving healthcare outcomes depends essentially on our ability to discover disease- and/or drug-associated clinically actionable genetic mutations. Integration and manipulation of diverse genomic data and comprehensive electronic health records (EHRs) on a big data infrastructure can provide an efficient and effective way to identify clinically actionable genetic variants for personalized treatments and reduce healthcare costs. We review bioinformatics processing of next-generation sequencing (NGS) data, bioinformatics infrastructures for implementing precision medicine, and bioinformatics approaches for identifying clinically actionable genetic variants using high-throughput NGS data and EHRs.

## 1. Introduction

High-throughput genomics technology has made possible the era of precision medicine, an approach to healthcare that involves integrating a patient's genetic, lifestyle, and environmental data and then comparing these data to similar data collected for thousands of other individuals to predict illness and determine the best treatments. Precision medicine aims to tailor healthcare to patients by using clinically actionable genomic mutations to guide preventive interventions and clinical decision making [1]. In the past 25 years, more than 4,000 Mendelian disorders have been studied at the genetic level [2]. In addition, more than 80 million genetic variants have been uncovered in the human genome [3, 4]. Clinical pharmacology research using electronic health record (EHR) systems has recently become feasible as EHRs have been implemented more widely [5]. Also, studies such as the Electronic Medical Records and Genomics-Pharmacogenomics (eMERGE-PGx) project [6], GANI_MED project [7], SCAN-B initiative [8], and Cancer 2015 study [9] have been designed to assess the value of next-generation sequencing (NGS) in healthcare.

Combining the functional characterization of identified genomic mutations with comprehensive clinical data available in EHRs has the potential to provide compelling evidence to implicate novel disease- and/or drug-associated mutations in phenotypically well-characterized patients. NGS is increasingly used in biomedical research and clinical practice. NGS technological advances in clinical genome sequencing and adoption of EHRs will pave the way to create patient-centered precision medicine in clinical practice. NGS technology is an essential component supporting genomic medicine but the volume and complexity of the data pose challenges for its use in clinical practice [10]. Sequencing a single human genome generates megabytes of data; therefore, investment in a bioinformatics infrastructure is required to implement NGS in clinical practice.

The term “big data” is defined differently by different people [11]. Gartner defines big data as “high-volume, high-velocity, and/or high-variety information assets that demand cost-effective, innovative forms of information processing that enable enhanced insight, decision making, and process automation” (http://www.gartner.com/it-glossary/big-data/) while others define it as the 5 Vs, which are Volume, Velocity, Variety, Verification/Veracity, and Value [12]. In this review, we describe how one source of big data, in the form of genomic data generated by NGS, is processed and being used to improve healthcare and clinical research. We give an overview of NGS technologies, bioinformatics processing of NGS data, bioinformatics approaches for identifying clinically actionable variants in sequence data, guidelines for maintaining high standards when generating genomic data for clinical use, bioinformatics infrastructures of studies aimed at implementing precision medicine, and methods for ensuring the security of genomic data. We also discuss the need for the efficient integration of genomic information into EHRs.

## 2. Genomic Data Generation

### 2.1. Approaches to Sequencing

NGS includes DNA sequencing and RNA sequencing (RNA-seq) (Table 1). DNA sequencing approaches include (1) whole-genome sequencing (WGS), (2) whole exome sequencing (WES) of the coding regions of all known genes, and (3) targeted sequencing of genomic regions or genes implicated in a disease [13]. In addition, RNA-seq is used in transcriptome profiling to sequence all RNA transcripts (the transcriptome) in cells at a given time point to measure gene expression, targeted sequencing for measuring the expression of transcripts encoded by a specific genomic region, and sequencing of small RNAs. Targeted DNA sequencing is already being applied in some areas of clinical practice such as pharmacogenomics (e.g., the eMERGE-PGx project [6]), while WGS, and particularly WES, is emerging into the clinic for the evaluation of developmental brain disorders such as intellectual disability [14], autism [15], and seizures [16]. With continuing decreases in the costs of sequencing, it is expected that the use of WES/WGS and RNA sequencing in healthcare will become more common.

### 2.2. Read Depth

NGS involves breaking DNA into fragments and determining the order of the nucleotide bases in each fragment. The sequence of each fragment is called a “read.” Because the distribution of reads across the genome is uneven (due to biases in sample preparation, sequencing-platform chemistry, and bioinformatics methods for genomic alignment and assembly of the reads) [17, 18], some bases are present in more reads and others in fewer reads. Read depth refers to the number of reads that contain a base; for example, a 10x read depth means that each base was present in an average of 10 reads. For RNA-seq, read depth is more often stated in terms of the number of millions of reads. Variant calling is more reliable with increasing read depth, and a greater depth is advantageous for detecting rare genetic variants with confidence. The read depth needed can depend on multiple factors including guidelines from the scientific community, the presence of repetitive genomic regions (these are more difficult to sequence), the error rate of the sequencing platform, the algorithm used for assembling reads into a genomic sequence, and gene expression level (for RNA-seq). Read depth recommendations from the scientific literature include 100x for heterozygous single nucleotide variant detection by WES [19], 35x for genotype detection by WGS [20], 60x for detecting insertions/deletions (INDELs) by WGS [21], 10–25 million reads for differential gene expression profiling by RNA-seq [22], and 50–100 million reads for allele-specific gene expression by RNA-seq [23].

### 2.3. Sequencing Technologies

#### 2.3.1. Description of Technologies

Commercially available sequencing platforms use a variety of methods to generate sequence data (Table 2). Sequencing-by-synthesis (MiSeq and HiSeq 4000 platforms) is the enzymatic synthesis of a DNA strand complementary to a template DNA strand. For NGS, the procedure involves DNA fragmentation, creation of a DNA library by attaching adaptors to each fragment, amplification of the fragments on a solid surface, synthesis of a DNA strand complementary to each template DNA fragment (using DNA polymerase), and fluorescence imaging to identify each newly incorporated nucleotide on the synthesized DNA strands [24]. Single-molecule, real-time sequencing (PacBio RS II platform) is a modification of sequencing-by-synthesis [25]. In this approach, each DNA polymerase molecule is immobilized at the bottom of a nanoscale well called a zero-mode waveguide. A laser light illuminates the well from below and emits a pulse of light when a fluorescent-labelled nucleotide is added to the nascent DNA strand by DNA polymerase (bound to a template DNA fragment), allowing detection of the incorporated nucleotide. Semiconductor sequencing (Ion S5 platform) is another modification of sequencing-by-synthesis that uses a semiconductor-sensing device to detect the addition of unmodified nucleotides during DNA synthesis [26]. Pyrosequencing (454 GS FLX Titanium XL+ platform) is a technique that couples sequencing-by-synthesis to a chemiluminescent enzyme (luciferase) reaction that generates visible light allowing detection of nucleotide incorporation during DNA synthesis [27]. Oligonucleotide ligation (SOLiD 5500xl W platform) involves ligating oligonucleotide probes to template DNA strands to determine the sequence of the template [28]. Sequencing by dideoxynucleotide (ddNTP) chain termination (Sanger Genetic Analyzer 3500xL platform), often called Sanger sequencing, involves incorporation of ddNTPs by DNA polymerase during DNA synthesis [29]. Fluorescence labelling allows identification of each of the ddNTPs added to the synthesized DNA strands.

#### 2.3.2. Comparison of Sequencers

The MiSeq, PacBio RSII, and Ion S5 sequencers were designed for targeted sequencing and sequencing small genomes (e.g., the genomes of microorganisms) whereas the HiSeq 4000, 454 GS FLX Titanium XL+, and SOLiD 5500xl W can be used for WES and WGS of human genomes (Table 2). The instruments most often used in precision medicine programs performing WES/WGS of the human genome in clinical care settings are the HiSeq sequencers [30] that have the advantages of a relatively high sample throughput and a low sequencing error rate. However, all of the NGS technologies are being applied to health research [31–36]. The single-molecule, real-time sequencing technology generates the longest reads (Table 2), making the PacBio RS II instrument well suited for de novo sequencing (by assembly of reads into long contiguous sequences) of the genomes of organisms that do not have a reference genome (e.g., many microbial genomes) [37].

The sequencers that cost the least are the bench-top Ion S5 and MiSeq instruments (Table 2), and for many laboratories it would be feasible to buy more than one of these instruments. While they can be used to perform WES of the human genome, the sequencing cost per base would be much higher compared with WES on the HiSeq instrument. The HiSeq 4000, 454 GS FLX Titanium XL+, and SOLiD 5500xl W instruments are more expensive, costing between $500,000 and $900,000 each, but they are capable of sequencing several human genomes or exomes within a few days to one week. Large laboratories that expect to assay many samples routinely by WES/WGS might consider it cost-efficient to buy more than one of these sequencers to meet assay demand. All six next-generation sequencers in Table 2 produce at least 0.5 gigabases per run and most output several gigabases per run, giving an idea of the volume of data that needs to be considered when planning for the data storage and processing capabilities of bioinformatics pipelines to be used in clinical laboratories that perform NGS assays.

#### 2.3.3. Sequencing Accuracy

With continued refinement in technology, many NGS platforms have demonstrated a low rate of errors in variant detection (1/1000 to 1/50 bases depending on the instrument and read depth) [38, 39]. Previous reports have compared sequencing accuracy among the technologies presented in Table 2. In a comparison of the HiSeq 2000 and SOLiD 5500xl platforms for WGS of human DNA samples, the HiSeq 2000 had higher sensitivity for calling single nucleotide variants but the SOLiD 5500xl had a lower false positive rate [40]. When the Ion PGM, MiSeq, and PacBio RS sequencers were compared by sequencing four microbial genomes, the PacBio RS had the highest sequencing error rate, and Ion PGM data had slightly more variant calls and a higher false positive rate than MiSeq data [41]. Compared with other technologies, the 454 and PacBio RS platforms have demonstrated the most unbiased read distribution in genomic regions with a high GC content [41, 42], an important factor affecting the probability of calling a variant in these regions. However, the 454 platform has a tendency to assess the length of homopolymer tracts incorrectly, resulting in false positive single nucleotide variant calls in these tracts [42].

In comparison with NGS technologies, Sanger sequencing is widely considered the most accurate sequencing method (error rate as low as 1 in 10,000 bases) [43] and remains the gold standard. Genetic variants detected using NGS should always be validated by an independent method if the variants are relevant to clinical care or are associated with health outcomes in research studies. Because of its high accuracy, Sanger sequencing is often used for validation. Other methods of validation, especially for common single nucleotide variants, INDELs, or structural variants, include polymerase chain reaction (PCR) and genotype/copy number variant arrays.

## 3. Genomic Data Processing and Quality Control

### 3.1. Data Processing

**Figure 1:**
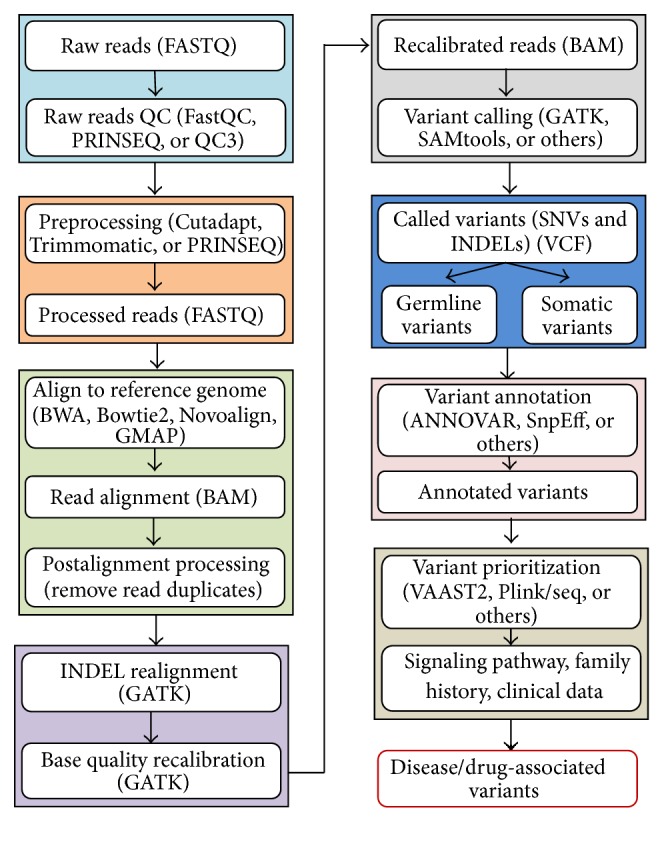
A flow chart of processing next-generation sequencing data.

Data files generated by next-generation sequencers contain raw sequence reads, each with a unique identifier, and their quality scores. Sequence reads need to be evaluated for data quality and to exceed minimum quality thresholds, before being processed for read alignment [44], variant calling [45], and variant annotation [46, 47] in a bioinformatics pipeline (Figure 1). Read alignment involves aligning the sequence reads to a reference sequence [48, 49] of the human genome to allow comparison of sequence data from the patient sample with the reference sequence. Reads with an uncertain alignment location need to be removed before further data processing. Alignment allows a number of quality control measures to be determined, for example, the percentage of all reads that align to a reference sequence, the percentage of unique reads that align to a reference sequence, and the number of reads that align at a specific locus (read depth). These measures influence the reliability of variant calling, the next step in a NGS bioinformatics pipeline. Variant calling tools, such as SAMtools [50], GATK [45], and others, are used to identify differences in sequence between the patient sample and a reference. These differences can include changes of one nucleotide (single nucleotide variants, SNVs), a few nucleotides (small INDELs), or larger regions, such as copy number variants (CNVs) and other structural variations (SVs). These software programs allow users to specify different parameters to adjust for minimizing false positive and false negative variant calls. Variant annotation depends on biological knowledge and provides information on the known or likely impact of variants on gene and protein function [46, 47]. To produce a patient report, annotated variants are interpreted in a disease-specific context and are often classified based on their known or expected clinical impact. For instance, the ClinVar [51] variant database, released on May 4, 2015 (http://www.ncbi.nlm.nih.gov/clinvar/), by the National Center for Biotechnology Information (NCBI), contained more than 110,000 unique genetic variants having clinical interpretations [52].

### 3.2. Clinically Actionable Variants

**Figure 2:**
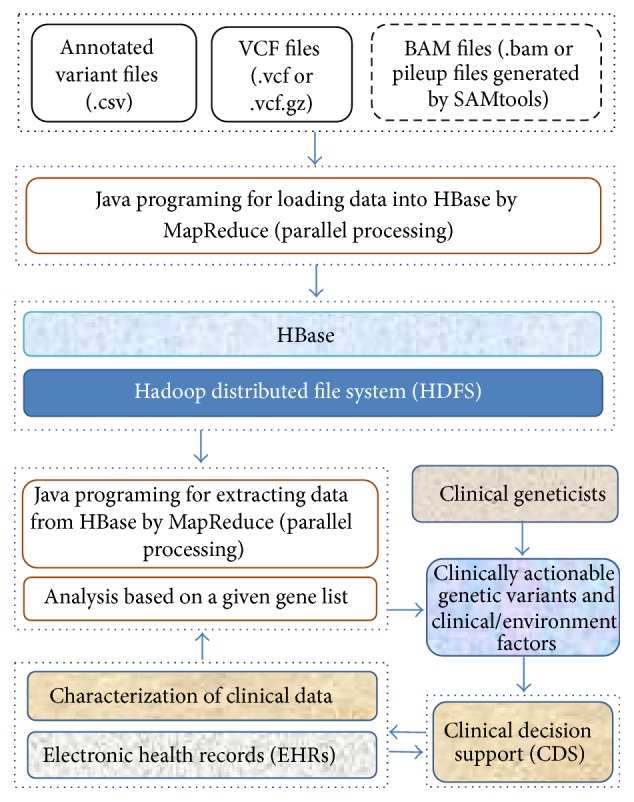
The basic framework of SeqHBase for detecting clinically actionable genetic variants.

In clinical care, the American College of Medical Genetics and Genomics (ACMG) has recommended the identification and return of incidental findings (IFs) for clinically significant variants in a set of 56 “highly medically actionable” genes associated with 24 inherited conditions [53, 54]. Also, the National Heart, Lung, and Blood Institute (NHLBI) Exome Sequencing Project (ESP) has reported actionable exomic IFs from 112 genes in 6,503 participants [55]. The 112 genes included 52 ACMG genes and an additional 60 “actionable” genes. To infer biological insights from massive amounts of NGS data and comprehensive clinical data in a short period of time, we have developed an analysis pipeline within a software framework called SeqHBase [56] (Figure 2) to quickly identify disease- or drug-associated genetic variants. There were more than 27 million unique variants among 300 patients with WGS data that we analyzed using SeqHBase. In addition to identifying variants that are annotated as “pathogenic” or “likely pathogenic” by ClinVar [51], we compiled a list of low frequency or rare variants that are possibly damaging, and novel loss-of-function (LoF) variants that are absent in the ClinVar database, to allow clinical geneticists to review the potential pathogenicity of these variants further. As SeqHBase is a big data-based toolset, it takes only a few minutes to analyze WGS data for 300 individuals and to generate a candidate list of actionable genomic variants. More detailed discoveries from these WGS data will be described in future reports.

SeqHBase is one of several, freely accessible bioinformatics tools for prioritization of variants from WES/WGS data. Daneshjou et al. reported a web-based tool for identifying clinically actionable variants in the 56 ACMG genes [57], and Zhou et al. developed a variant characterization framework for targeted analysis of relevant reads from high-throughput sequencing data [58]. Other tools include PHIVE [59] which prioritizes variants in genes responsible for mouse model phenotypes that are similar to the phenotypes of patients being tested by WES and OVA [60] that performs prioritization by integrating data on human and model organism phenotypes, gene function, and known biological pathways.

Identifying clinically actionable variants remains a challenge despite the availability of variant prioritization tools. A workshop convened by the National Human Genome Research Institute and the Wellcome Trust identified limited evidence of the clinical significance of genetic variants and the lack of a comprehensive database of genetic variant-phenotype associations as barriers to the implementation of precision medicine [61]. It was noted that existing catalogs of clinically actionable variants are not standardized, are maintained by different entities (e.g., laboratories or government organizations), and are not designed to interact with EHRs. To speed the incorporation of genomic data into clinical care, the workshop advocated for a dynamic, centralized database that can be updated with available, reliable evidence on variant pathogenicity. The Clinical Genome Resource (ClinGen) program [52], developed in response to this recommendation, provides resources (e.g., ClinVar [51]) to aid the understanding of genetic variation and the use of genetic variation in clinical practice.

### 3.3. Quality Control

Best practices for quality control in the bioinformatics processing of NGS data have been reported in the scientific literature [45, 62]. Quality control metrics include total reads, ratio of unique reads to total reads, proportion of bases covered at a specified minimum read depth, mean read depth, raw sequence error rates, ratio of transitions (pyrimidine-to-pyrimidine or purine-to-purine mutation) to transversions (pyrimidine-to-purine mutation or vice versa), missingness (proportion of genomic sites at which a variant could not be called), homozygosity, heterozygosity, and distribution of known and novel variants relative to those contained in the dbSNP database. For targeted or exome sequencing, an additional metric is capture efficiency, the percentage of targeted bases that are covered by one or more reads.

These metrics can be calculated using the PLINK/SEQ (https://atgu.mgh.harvard.edu/plinkseq/) or VCFtools [63] software programs that can readily be incorporated into a bioinformatics pipeline, allowing assessment of NGS data quality in both clinical and research settings. Values for the first four metrics depend on the type of sequencing assay performed but, in general, higher values indicate better data quality. The raw sequence error rates and missingness should be as low as possible. The ratio of transitions to transversions (Ti/Tv ratio) is expected to be ~2.0–2.1 for WGS data overall, 2.10 for known variants in WGS data, 2.07 for new variants in WGS data, ~3.0–3.3 for WES data overall, 3.5 for known variants in WES data, and 3.0 for new variants in WES data [45]. Homozygosity and heterozygosity depend on the type of population: heterozygosity is expected to be more frequent in admixed populations and homozygosity to be more frequent in inbred populations. It is estimated that each person has ~200 novel SNPs not present in the dbSNP database [64]; therefore, a value that is much larger than 200 is indicative of a high false positive rate of single nucleotide variant calls. Capture efficiency is reported to range within ~50–75% [65].

There are no existing, quality control standards that relate to generating clinical interpretations for genetic variants. However, substantial efforts are being made to identify clinically actionable pharmacogenetics variants, and it is instructive to review the approach being used. The Coriell Personalized Medicine Collaborative [66], the Clinical Pharmacogenetics Implementation Consortium [67], the Pharmacogenetics Working Group established by the Royal Dutch Association for the Advancement of Pharmacy [68], and the Evaluation of Genomic Applications in Practice and Prevention initiative sponsored by the Centers for Disease Control and Prevention [69] have independently developed similar processes for selecting candidate drugs, reviewing the published literature to identify drug-gene associations, scoring the evidence supporting associations between genetic variants and drug response, and interpreting the evidence to provide treatment guidelines.

This approach involving review and interpretation of the scientific literature by an expert committee can be considered the gold standard for determining whether a variant is clinically relevant or actionable but also can be expensive and time-consuming. It will not be feasible for experts, either individually or in committees, to review the large number of genetic variants identified in NGS data. Tools such as POLYPHEN-2 [70], VEP [71], MutationAssessor [72], and SIFT [73] can be used to predict variant effects. However, because these tools are sometimes inaccurate [74] and often differ in their predictions for the same variant [75, 76], there will likely be many variants that have no clear predicted, clinical interpretation. Furthermore, an additional problem is that the predictions made by these tools are not specific to a given gene or class of genes. For example, many genes would tolerate the substitution of glycine for another amino acid, but, in a gene that encodes a collagen fibril, loss of a glycine would impair fiber assembly resulting in a significant phenotype [77]. New methods that are both accurate and efficient need to be developed for predicting the pathogenicity of genetic variants found by NGS.

A limitation of using the ClinVar database [51] to identify clinically actionable genomic mutations is that a genetic variant in ClinVar can be described as having a different potential for pathogenicity by different submitters. For example, of the 12,895 unique variants with multiple clinical interpretations that have been submitted by more than one laboratory, 2,229 (17%) were interpreted differently by different submitters, with one- or two-step differences between any of three major levels: “pathogenic or likely pathogenic,” “uncertain significance,” and “likely benign or benign” [52]. Differences in interpreting the pathogenicity of variants have also been reported by the Clinical Sequencing Exploratory Research (CSER) program [30], an initiative designed to trial the use of WES/WGS data in clinical practice. The program compared CSER laboratories on their clinical interpretations of 98 variants and observed one-step differences in interpretation for 42% of variants and two-step or larger differences for 20% of variants [78]. To estimate and interpret the pathogenicity of new variants that are absent in the ClinVar database and to achieve some level of consensus on the clinical interpretations of variants, evaluations from experts, such as clinical geneticists, and/or further biological functional studies are needed.

## 4. Guidelines for Bioinformatics Processes

### 4.1. Summary of Guidelines

Bioinformatics pipelines are constituted of multiple databases and software programs to convert raw sequence reads to a list of clinically actionable or candidate variants. To promote the transparency of pipeline processes and data flow, the ACMG [79], the College of American Pathologists (CAP) [80], Weiss et al. [81], and Gargis et al. [82] have offered guidelines for NGS and the operation of bioinformatics pipelines in a clinical setting. The recommendations of these guidelines include thorough documentation of the pipeline and of deviations from pipeline standard operating procedures (e.g., software updates, changes in software settings, operator error, hardware failure, or other failures in the pipeline), validation of the pipeline, development of a pipeline quality management program, and implementation of policies to ensure secure data storage and data transfer.

The recommendations for written patient reports state that gene names should be provided according to HUGO Gene Nomenclature Committee nomenclature (http://www.genenames.org/) and genetic variants according to the nomenclature guidelines of the Human Genome Variation Society (http://www.hgvs.org/). Laboratories should follow the recommendations of the ACMG [53, 54] for interpreting the clinical significance of variants. Patient reports should also include the genome build and reference sequence used for variant detection, the genomic coordinates of identified variants, and mention of whether clinically significant variants were confirmed by an independent assay method [81]. Laboratories should also report genetic variant data (gene name, zygosity, cDNA nomenclature, protein nomenclatures, exon number, and clinical significance) in a structured format according to HL7 standards (HL7 version 2 Implementation Guide: Clinical Genomics, http://www.hl7.org/implement/standards/). This is aimed at providing sufficient data to facilitate both clinical decision support and the display of genetic information in the EHR.

Challenges to implementing these guidelines include the constantly evolving nature of NGS technologies, bioinformatics tools (necessitating frequent updates of the bioinformatics pipeline), clinical interpretation (necessitating frequent updates of genetic variant annotation), the limited capacity of health care organizations/laboratories to store the voluminous data generated by NGS platforms (data storage options considered must ensure security of the stored genetic data), and the need for personnel trained in bioinformatics and statistics to develop a bioinformatics pipeline and to process and analyze NGS data. However, these challenges are not insurmountable, and it is likely that health care institutions that want to use NGS data in clinical care will attempt to overcome these hurdles and follow the guidelines.

### 4.2. Accreditation from the College of American Pathologists (CAP)

**Figure 3:**
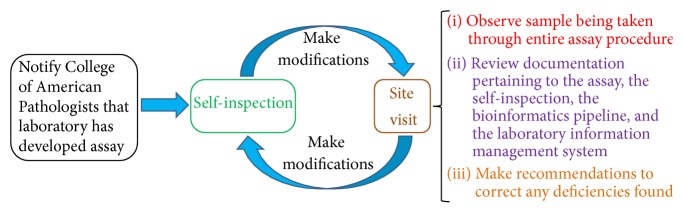
Overview of steps for a laboratory to obtain accreditation by the College of American Pathologists.

Clinical laboratories that develop Clinical Laboratory Improvement Amendments- (CLIA-) certified NGS assays based on CAP standards [80] can seek accreditation from CAP, an agency that can provide accreditation on behalf of the CLIA program. The accreditation process involves a site visit inspection by a peer institution/laboratory once every two years and a self-inspection in alternate years (Figure 3). For the self-inspection, CAP sends the laboratory a list of items, specific to the NGS assay, that need to be checked by the laboratory. Completing the self-inspection for a NGS assay would allow the laboratory to determine how closely it adheres to the CAP standards for the assay. For the site visit, the inspectors would observe a sample being taken through the entire assay procedure. Any deficiencies found must be corrected, and CAP should be provided with a report describing the corrective measures within 30 days after the site visit. Through the mechanism of CAP accreditation, the laboratory would inform external entities that it provides a CLIA-certified assay that meets CAP standards for the assay.

## 5. Bioinformatics Infrastructure for Genomic Data

### 5.1. Separate Databases for Different Types of Data

**Figure 4:**
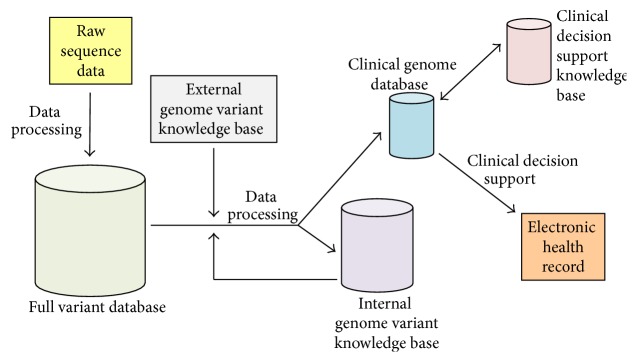
Elements of a proposed infrastructure for bioinformatics processing of sequencing data in clinical laboratories.

Welch et al. have proposed an infrastructure comprised of independent, interacting databases for processing and storing genomic data in a clinical setting [83] (Figure 4). These databases include a “full variant database” to store all genetic variants for each patient, a clinical genome database to store only the clinically relevant variants for each patient, a clinical decision support knowledge base that integrates decision rules and guidelines for providing care (e.g., drug dosing rules) with genomic and clinical information, and a genome variant knowledge base to store known genetic variants and their clinical interpretations. ClinVar [51] is an example of a freely accessible genome variant knowledge base but clinical laboratories will likely also maintain their own internal genome variant knowledge bases (based on the genomic data of patients they test). The proposed infrastructure can potentially accommodate large amounts of genomic data because it involves warehousing the data external to EHRs. However, it would require investment in data storage capacity external to the EHR database system and the development and maintenance of interfaces between the genomic databases and the EHR database system [84].

### 5.2. Cloud Computing

**Figure 5:**
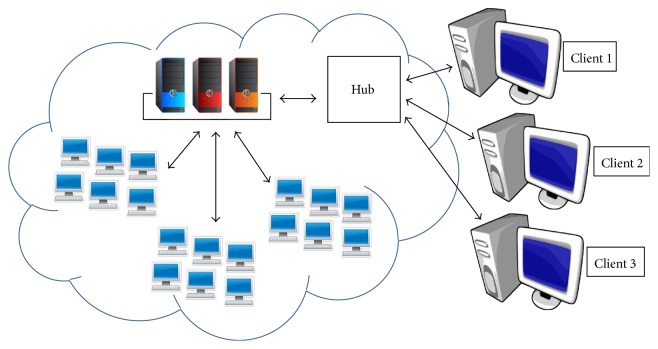
Cloud computing diagram.

Cloud computing, involving the use of remote servers to store and access data and software programs (Figure 5), has also been proposed for genomic data processing and storage. Cloud computing providers offer infrastructure, software, and programming platforms as services and incur the costs for developing and maintaining these services [85]. Because clients pay only for the services they use, cloud computing offers an economical approach to genomic data management compared with investment in the creation and maintenance of databases by healthcare entities to house genomic data. Hadoop is an open-source programming platform that is already being used to develop software for genomic data processing in a cloud computing environment [85]. Hadoop breaks data into small fragments, distributes the fragments over many computers, distributes computation to where the fragments are located so all fragments are processed in parallel, and aggregates the results at the end of computation [85]. The parallel processing of many small pieces of data greatly reduces computation time. Examples of open-source software developed on the Hadoop platform for processing genomic data are Crossbow [86], GATK [87], and Hadoop-BAM [88]. Challenges to the use of cloud computing for genomic data include the long data transfer times for uploading NGS data files to the cloud, the perceived lack of data security in the cloud computing environment, and the need for advanced programming skills in Java to develop software using Hadoop [85].

### 5.3. Infrastructure for Data Sharing

The separation of genomic and clinical data repositories facilitates the use of genomic data in research as well as clinical care. To engage in collaborative research, infrastructure for sharing genomic data with researchers internal and external to the institution that generated the data is required. The Global Alliance for Genomics and Health (GA4GH) [89], an international coalition of healthcare and academic centers that aims to advance the sharing of genomic and clinical data to improve health, has launched efforts to create such an infrastructure. The group has developed an application programming interface (API) to support the sharing of data on DNA sequences and genomic variants across organizations and bioinformatics pipelines [90]. GA4GH is also developing APIs for other types of genomics-related data including variant annotations, RNA-seq, and genotype-phenotype associations. These tools will allow genomic data from multiple organizations to be analyzed in aggregate, increasing statistical power to identify genetic variants that have a clinical effect.

### 5.4. Security of Genomic Data

Genomic data is protected health information; therefore, its privacy and confidentiality should be maintained similarly to other protected health information. Safeguards include the use of data encryption, password protected files, secure data transfer, audits of data transfer processes, and the implementation of institutional policies against data breeches and malicious use of the data [91]. The use of cloud computing presents added security concerns because data storage and/or processing services are provided by an entity external to the healthcare organization. Measures that the cloud service provider can take to address these concerns include logging access to the data, creating a role-based access system (level of access depends on the type of user), complying with third-party certifications for information security (e.g., the International Organization for Standardization/International Electrotechnical Commission 21001:2013 information security standard http://www.iso.org/iso/home/standards/management-standards/iso27001.htm/), protecting the security of the computer network, using notification alarms to track when changes are made to stored data, and guaranteeing the complete removal of data from its servers once the cloud storage service is no longer being used [92].

## 6. Examples of Implementing Genomic Data in Clinical Care

### 6.1. Clinical Sequencing Exploratory Research Program

A survey of six health centers participating in the CSER consortium has described how the centers have integrated genomic data into the EHR [30]. Five centers performed sequencing at their own laboratories, and one site used an external laboratory but confirmed variants on-site using Sanger sequencing. Each center created a local bioinformatics pipeline for variant annotation, but all used multiple online catalogs of variants (e.g., ClinVar [51] and dbSNP) for annotation. Each site also built and maintained its own genome variant knowledge base (based on genetic variants ascertained in patients at the site) and created tools to use data from this internal database in variant annotation. Additionally, sites used manual or semiautomated methods to search the scientific literature or online gene-specific databases to determine the clinical significance of variants. EHR software was obtained from commercial providers at four centers and was locally developed at two centers. The laboratories at all six centers generated a human-readable PDF document, containing genetic results, that was designed to be incorporated into the EHR. The two sites with custom-built EHRs, and one site with commercial EHR software, also reported results in a structured, machine-readable format. Active clinical decision support (automated alerts through the EHR) for genetic variants was available at two of the centers. Only one center had an automated system for sending alerts to physicians when new genomic findings resulted in the reclassification of a genetic variant's clinical significance (e.g., a variant initially classified as being of unknown significance was subsequently discovered to have serious clinical consequences).

### 6.2. eMERGE Network Pharmacogenetics Study

Sites in the eMERGE network are also engaged in pilot efforts to incorporate genomic data, particularly data relevant to pharmacogenetics, into EHRs [6]. At one eMERGE site, separate data repositories were created for unprocessed sequence/genotype data and for variants of known pharmacogenetics relevance [93]. Software that applied approved pharmacogenetics-medication guidelines to patients' genetic data was used to determine a patient's pharmacogenetics phenotype (e.g., predicted poor metabolizer of a specific drug), and the phenotype data were stored as a laboratory result in the EHR. The site developed software that extended its existing, custom-built medication alert system, enabling the system to check for a relevant pharmacogenetics laboratory result when a physician prescribes a pharmacogenetics-related drug. If a patient has a pharmacogenetics phenotype, the system sends an alert to the physician and suggests alternative treatment. Another eMERGE site reported developing similar infrastructure that supported storage of all genetic variants separately from variants with pharmacogenetics relevance, the translation of genetic data into genotype-phenotype associations, and active clinical decision support for physicians prescribing pharmacogenetics-related drugs [94]. Changes in the clinical interpretation of genetic variants (based on new knowledge) that resulted in phenotype reassignment prompted the site to update its genotype-phenotype translation database to reflect the newly determined genotype-phenotype relationships. Because this database was linked to the site's clinical information system, pharmacogenetics data in the EHR was automatically updated.

### 6.3. Lessons from CSER and eMERGE

The CSER and eMERGE pharmacogenetics programs are in progress and have not yet reported on improvements in patient outcomes as a result of incorporating genomic data into clinical care. Each site in these programs had its own customized bioinformatics pipeline, laboratory information management system, clinical decision support capabilities, and electronic health records that would not be generalizable to other sites. This presents a challenge as a more uniform infrastructure for genomic data processing could be adopted more widely and easily. Based on their experiences, sites in both programs identified a number of factors that need to be addressed to facilitate the integration of genomic data into healthcare: (1) the requirement for active clinical decision support; (2) tools to examine and interpret sequence variants, especially new, undefined variants; (3) approaches to update changes in the clinical significance of sequence variants over time; (4) giving healthcare providers access to consultants trained in genetics; (5) infrastructure for secure and reliable delivery of results to external healthcare providers; and (6) methods for explaining genomic information to patients.

## 7. Discussion

The ideal, preventive model of patient care is to understand as much about a patient as possible, as early in his/her life as possible, to detect warning signs of serious but preventable illness at an early stage so that preemptive health interventions can be simpler and/or less expensive than treatment implemented at a later stage. Also, knowing a person's individual characteristics is often relevant for providing effective treatment against disease because patients can respond differently to the same treatment. By facilitating precision medicine, advances in genomics have the potential to change the way we prevent and treat diseases. However, the translation of these advances into reality for patient care depends mainly on our ability to discover disease- and/or drug-associated clinically actionable genetic mutations and on our understanding of the roles of these mutations in the disease process.

Healthcare centers that are conducting pilot studies of the integration of genomic data into clinical care have developed a bioinformatics infrastructure for processing NGS data that consists of a group of databases ancillary to the EHR [30, 93, 94]. The infrastructures were, for the most part, locally developed and proprietary, but this is because these centers are among the first healthcare providers to use genomic data in clinical care and there are no established infrastructures to meet their bioinformatics needs. The infrastructures were built along the same general plan: a bioinformatics pipeline for processing NGS data, a database for storing all genetic variants detected in patient samples, a genome variant knowledge base for storing known genetic variants and their clinical interpretation, a database for the subset of variants deemed to be clinically actionable (with variants linked to a specific clinical phenotype), links between databases allowing data transfer, and a method for reporting the results of clinically actionable variants in the EHR. Developing and maintaining a bioinformatics infrastructure for NGS data requires substantial investment in resources and personnel and can be too expensive for small clinical laboratories. However, because genetic variant databases are maintained separately from the EHR, it might be possible for multiple, small laboratories to pool resources to build and share a common bioinformatics infrastructure. The storage and bioinformatics processing of raw NGS data output by sequencing platforms might exceed the infrastructure capacity of even some large healthcare organizations. Therefore, healthcare providers might want to consider cooperatively establishing a cloud computing service designed to store and process genomic data securely for the healthcare community. Clinical laboratories must also consider the cost of sequencing instruments as part of infrastructure costs. Bench-top instruments used for targeted sequencing are less expensive and output less data than instruments that perform WES/WGS. For these reasons, more laboratories are likely to perform targeted sequencing before, or instead of, attempting to build infrastructure to support WES/WGS.

A major challenge to incorporating genomic data into clinical care is the lack of standards for generating NGS data, bioinformatics processing, data storage, and clinical decision support. Standards would promote consistency in data quality, and adherence to standards would facilitate the routine use of genomic data in clinical practice, but it is difficult to create standards when NGS technology and bioinformatics software are constantly evolving. Further, approaches to clinical decision support vary across healthcare institutions [30]. In a survey of 17 health centers participating in the CSER program or the eMERGE network, most centers did not have active clinical decision support for genetic data in the EHR although there were existing mechanisms for clinically actionable information to trigger alerts in the majority of the EHR systems [95]. Centers with active clinical decision support either built their own software locally or customized the clinical decision support capabilities of commercial EHR software [30]. Most centers reported that genetics results were available as a portable document format (PDF) file in the EHR and recommended the development of clinical decision support for disease-defining and pharmacogenetics variants and creation of a clinical decision support knowledge base to advise on appropriate clinical actions (e.g., a change in treatment).

Appropriately integrating EHRs with genomic data for the discovery of clinically actionable variants can generate new insights into disease mechanisms and provide better predictions about effective treatments, all leading to improved targeting of interventions to patients. To generate knowledge on the nature of disease from comprehensive EHR data, new methods such as machine learning, natural language processing, and other artificial intelligence methods are needed. However not all patients are likely to benefit from the use of big data in healthcare due to our current knowledge gaps on how to extract useful information from large-volume genomic and clinical data and how to interpret discovered genetic variants appropriately. At the same time, targeted therapies are not yet available for many important genes, and regulatory issues need to be resolved before some useful bioinformatics tools can be applied in a healthcare setting.

Finally, as EHRs are extremely personal, measures to protect patient data have to be put in place to make certain that patient information is only shared with those who need to see it. Despite this challenge, the potential advantages that genomic data can bring to healthcare far outweigh the potential disadvantages. The growing trend towards integration of genomic data and EHRs will cause concern, but as long as patient privacy and data security can be rigorously maintained, genomic data is certain to play an essential role in precision medicine.

## 8. Conclusion

To reach the goal of precision medicine, healthcare institutions need to invest in a bioinformatics infrastructure and in personnel trained in bioinformatics and genetics, to develop the capacity to process, store, and interpret genomic data and to link these data with EHRs. In addition, more efforts are needed to distinguish genetic variants that are truly clinically actionable; that is, the variants are useful for guiding clinical decisions regarding interventions to improve health outcomes. Clinical research studies of the implementation of genomic data in healthcare can provide valuable lessons about how genomic data should be managed, and patient privacy protected, when incorporating genomic data into clinical practice on a larger scale. These lessons can alert healthcare institutions to the scientific and technical challenges of using genomic data in precision medicine.

## Figures and Tables

**Table 1:** Sequencing assays.

Characteristic	DNA sequencing			RNA-seq	
	Targeted genomic regions	Whole exome	Whole genome	Targeted	Transcriptome profiling
Capture method^*∗*^	Amplicon-based targeting; hybrid capture; in-solution capture	Hybrid capture; in-solution capture	None	Hybridization only; hybridization and extension; multiplexed PCR	None
Amount of genome/transcriptome sequenced	~150 bp–62 Mb (≤2% of genome)	~30–60 Mb (1-2% of genome)	~3 Gb (≥95% of genome)	Variable: transcripts of ~10–1000 genes	Entire transcriptome
Amplification	Yes	Yes	Not required	Yes	Required for low-quantity RNA samples
Sequencing depth	100–1000x^Ü^	80–100x^Ü^	30–50x^Ü^	0.3–25 million reads^‡^	15–200 million reads^‡^
Amount of sequence data generated per sample	~0.3–5 Gb	~4-5 Gb	~90 Gb	~0.5–3 Gb	~5-6 Gb

bp, base pairs; Mb, megabases; Gb, gigabases; PCR, polymerase chain reaction.

^*∗*^Method used to select genomic regions for sequencing.

^Ü^Number of times a single base is read during a sequencing run.

^‡^A greater number of reads are needed to detect rare transcripts.

**Table 2:** Comparison of sequencing instruments.

Characteristic	MiSeq	PacBio RS II	Ion S5	HiSeq 4000	454 GS FLX Titanium XL+	SOLiD 5500xl W	Sanger Genetic Analyzer 3500xL
Instrument price	~$125 K	~$695 K	~$65 K	~$900 K	~$500 K	~$595 K	~$173 K

Sequencing mechanism	Sequencing-by-synthesis	Single-molecule, real-time sequencing	Semiconductor sequencing	Sequencing-by-synthesis	Pyrosequencing	Oligonucleotide ligation	Dideoxynucleotide chain termination

Sequencing application	Targeted	Targeted; transcriptome profiling	Targeted; whole exome; transcriptome profiling	Whole exome/genome; transcriptome profiling	Whole exome/genome; transcriptome profiling	Whole exome/genome; transcriptome profiling	Next-generation sequencing validation, targeted sequencing of mutations or small insertions/deletions

Maximum read length	300 bp PE	10,000 bp	200 bp	150 bp PE	700 bp	75 bp SE, 50 bp mate-paired	850 bp

Reads per run	15 million	55–900 K	60–80 million	2.5–5 billion	~1 million	100 million–4.8 billion	Not applicable

Output data per run	0.5–15 Gb	0.5–16 Gb	~44 Gb	125–1500 Gb	~0.7 Gb	160–320 Gb	2–100 Kb

Run time	4–55 hours	6 hours	1-2 days	<1–3.5 days	23 hours	2–7 days	0.5–3 hours

Advantages	Low error rate; short run time	Long read length; short run time	Short run time; low start-up cost	Low error rate; high throughput	Long read length	Low error rate	Low error rate; long read length

Disadvantages	Higher cost per base compared to HiSeq instruments	Medium/high cost per base	High error rate for homopolymer tracts and insertions/deletions	Short read length	High error rate for homopolymer tracts	Short read length; long run time	High cost per base; low throughput

bp, base pairs; Gb, gigabases; K, thousand; Kb, kilobases; PE, paired-end; SE, single-end.
